# Immediate implant placement in molar extraction sites: a 1-year prospective case series pilot study

**DOI:** 10.1186/s40729-019-0201-7

**Published:** 2020-02-12

**Authors:** Henny J. A. Meijer, Gerry M. Raghoebar

**Affiliations:** 1 Department of Oral and Maxillofacial Surgery, University Medical Center Groningen, PO Box 30.001, NL-9700 RB Groningen, The Netherlands; 2 Department of Implant Dentistry, University Medical Center Groningen, Groningen, The Netherlands

**Keywords:** Immediate implant placement, Posterior region, Extraction sites, Implant survival

## Abstract

**Background:**

There is a growing tendency to place single tooth implants immediately after extracting a failing tooth in the posterior region. The aim of this prospective case series pilot study was to evaluate immediate implant placement in molar post-extraction sites during a 1-year follow-up period.

**Materials and methods:**

Fifteen consecutive patients with a single failing molar in the maxilla or mandible, and presenting enough bone to expect primary implant stability and an implant site free of infection, were included. The implants, with a large thread depth and sharp thread edges, were placed in each patient according to a two-staged surgical procedure. Three months later, a full contour screw-retained zirconia restoration with an angulated screw channel abutment was provided. Clinical and radiographic examinations were performed 1 month and 12 months after placing the restoration. In addition, the patients’ satisfaction with the restoration was scored after 12 months.

**Results:**

Four out of 15 of the mobile implants had to be removed before the 1-year evaluation. The implant and restoration survival rates were 73.3% at the 1-year evaluation (*n* = 15). The mean marginal bone loss, from loading to the 12-month follow-up, was 0.17 mm (*n* = 11). The mean plaque, calculus, peri-implant mucosa, bleeding, and pocket probing depth scores were low, depicting healthy peri-implant conditions. The patients were very satisfied.

**Conclusion:**

It was demonstrated, within the limitations of this study, that immediate placement of regular diameter implants in molar post-extraction sites in the maxilla and mandible resulted in a high implant failure rate during a 1-year follow-up period.

**Trial registration:**

Netherlands Trial Register, NL8117. Registered 24 October 2019 - Retrospectively registered, https://www.trialregister.nl/trial/8117.

## Background

Implant placement and loading protocols are changing [1]. There is a growing tendency to place single tooth implants immediately after the extraction of a failing tooth, especially in the maxillary aesthetic region, and preferably combined with immediate provisionalization [2–6]. This tendency is related to evolving society factors, including more demanding patients and a wish for direct treatment, whereupon innovations in implant surfaces and designs are facilitating the possibilities [7, 8]. Despite systematic reviews pointing out that there is a slightly higher risk of early implant loss compared to delayed implant placement, immediate implant placement in extraction sites is now presumed to be a reliable treatment option for single tooth implants [1, 2, 9].

It must be mentioned, however, that the majority of studies on immediate implant placement are related to the maxillary aesthetic region. Immediate implant placement in the posterior region is studied much less, probably because those patients are less demanding and due to the different anatomical features of the extraction socket compared to that of the single-rooted teeth in the anterior maxilla [10, 11]. Nevertheless, two systematic reviews mention that high survival rates have also been reported for immediate implant placement in molar regions [12, 13]. Although it was suggested that wide diameter implants may have better results in the molar region than regular diameter implants [13, 14]), they also were associated with a high failure rate [15]. Based on current published controlled studies, there is still a lack of evidence for an optimal immediate implant placement protocol in the molar region. Therefore, the aim of this prospective case series study was to evaluate immediate implant placement in molar post-extraction sites during a 1-year follow-up period.

## Materials and methods

### Patient enrolment

All patients referred to the Department of Oral and Maxillofacial Surgery (University of Groningen, University Medical Hospital), from January 2016 to July 2017, for single-tooth implant therapy in the maxillary and mandibular posterior region were considered for inclusion. The following inclusion criteria were applied:

- One failing first or second molar in the maxilla or mandible;
- Sufficient bone volume, with an intact buccal and lingual wall, to insert a dental implant of at least 7 mm in length;
- Implant site is free from infection;
- Adequate oral hygiene as expressed by the modified plaque index and the modified sulcus bleeding index from Mombelli et al. [16];
- Sufficient mesio-distal, bucco-lingual, and interocclusal space for the placement of an anatomic restoration;
- The patient is capable of understanding and giving informed consent.

Patients were excluded from the experimental protocol when at least one of the following exclusion criteria was met:

- Medical and general contra indications for the surgical procedures;
- Presence of active and uncontrolled periodontal disease;
- Bruxism;
- An active smoker;
- History of local radiotherapy to the head and neck region.

Patients fulfilling all the inclusion and none of the exclusion criteria were informed verbally and in writing about the study and signed the informed consent form.

The Medical Ethical Committee of the University Medical Center Groningen considered this case series study was not subject to the Medical Research Involving Human Subjects Act (Number M15.184100). The study was registered at the Netherlands Trial Register (Number NL8117).

### Surgical and prosthetic procedures

The surgical and prosthetic treatments were performed at the Department of Oral and Maxillofacial Surgery, University Hospital Groningen. One oral surgeon, experienced in implant dentistry, executed the surgical treatments and two experienced prosthodontists performed the restorative procedures. All the laboratory procedures were carried out in a single dental laboratory.

#### Surgical procedure

**Fig. 1:**
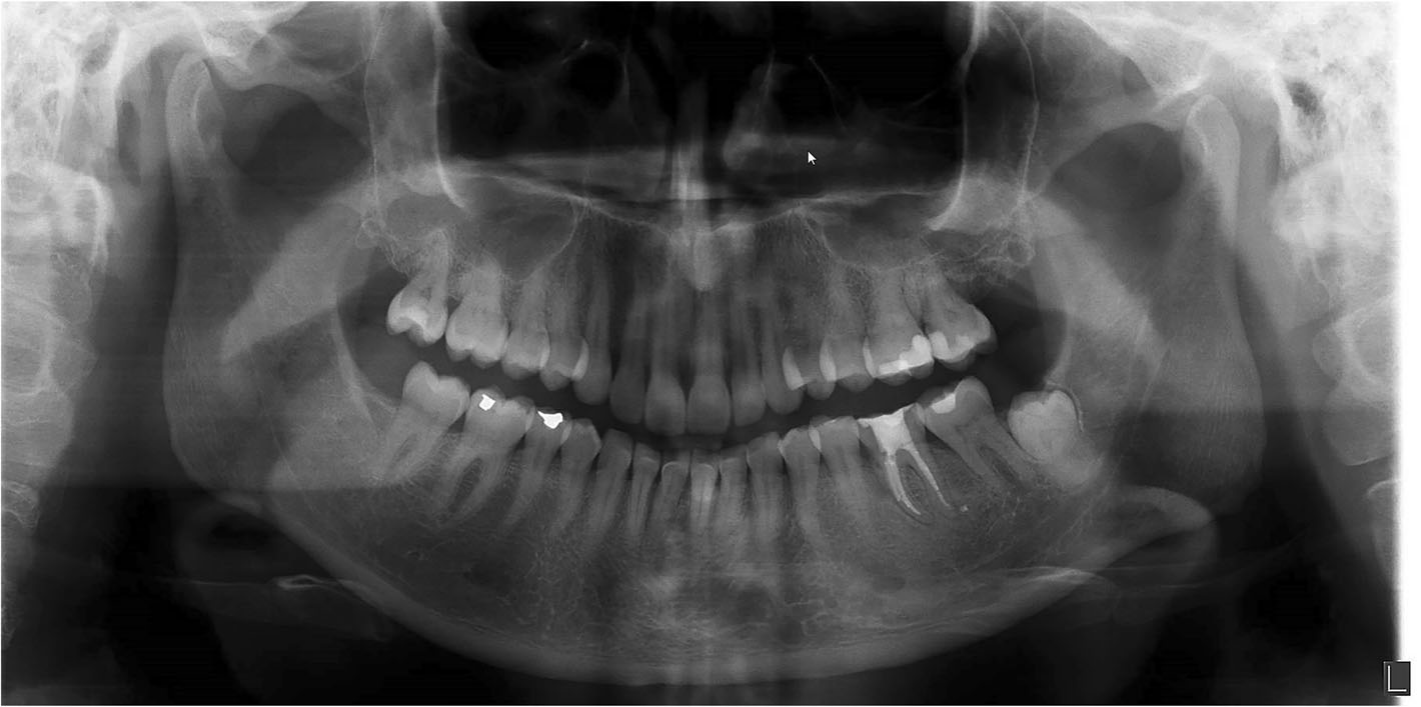
Pre-operative panoramic radiograph of a failing molar in position 36

The patients had a failing molar at the time of the intervention (Fig. 1). Antibiotic prophylaxis (2 g amoxicillin or, if allergic to penicillin, 600 mg clindamycin) was given 1 h pre-operatively as was a 0.2% chlorhexidine mouthwash (two times daily for 10 days) for oral disinfection. The first step of the surgical procedure, which was performed under local anaesthesia, involved carefully detaching the periodontal ligament from the failing tooth by an incision in the sulcus. Periotomes were used to extract the failing molar atraumatically. No mucoperiosteal flap was raised. The interradicular bone of the alveolus was prepared for the implant following the manufacturer’s protocol using a surgical template based on the ideal position of the prospective implant crown. The final twist drill was placed in the prepared socket. The remaining space between the drill and bone walls was augmented with a 1:1 mixture of autogenous bone, harvested from the retromolar or tuberosity area using a bonescraper (Bonescraper, Biomet 3i, Warsaw, Indiana, USA), and a bone substitute (Bio-Oss®, Geistlich Pharma AG, Wolhusen, Switzerland). The drill was carefully removed and a regular diameter implant (NobelActive, Nobel Biocare AB, Goteborg, Sweden) was placed, according to the manufacturers’ protocol. Regarding the corono-apical position of the implant, the shoulder of the implant was placed at a depth of 3 mm apical to the most apical aspect of the prospective clinical crown, with the help of a surgical template. The implant diameters were 4.3 mm, and the lengths varied from 8.5 mm to 10 mm, depending on the available bone height at the implant site. The primary implant stability was > 45 Ncm, measured with a manual torque wrench (NobelBiocare AB). A cover screw (NobelBiocare AB) was placed and the extraction socket closed with a mucosa graft, which was harvested from the tuberosity region. The wound was closed with Ethilon 5-0 nylon sutures (Johnson & Johnson Gateway, Piscataway, NJ, USA). One week after implant placement, a follow-up visit was scheduled for suture removal and to review the healing process. After 3 months, the implant was uncovered and a healing abutment (NobelBiocare AB) was installed.

#### Restorative procedure

**Fig. 2:**
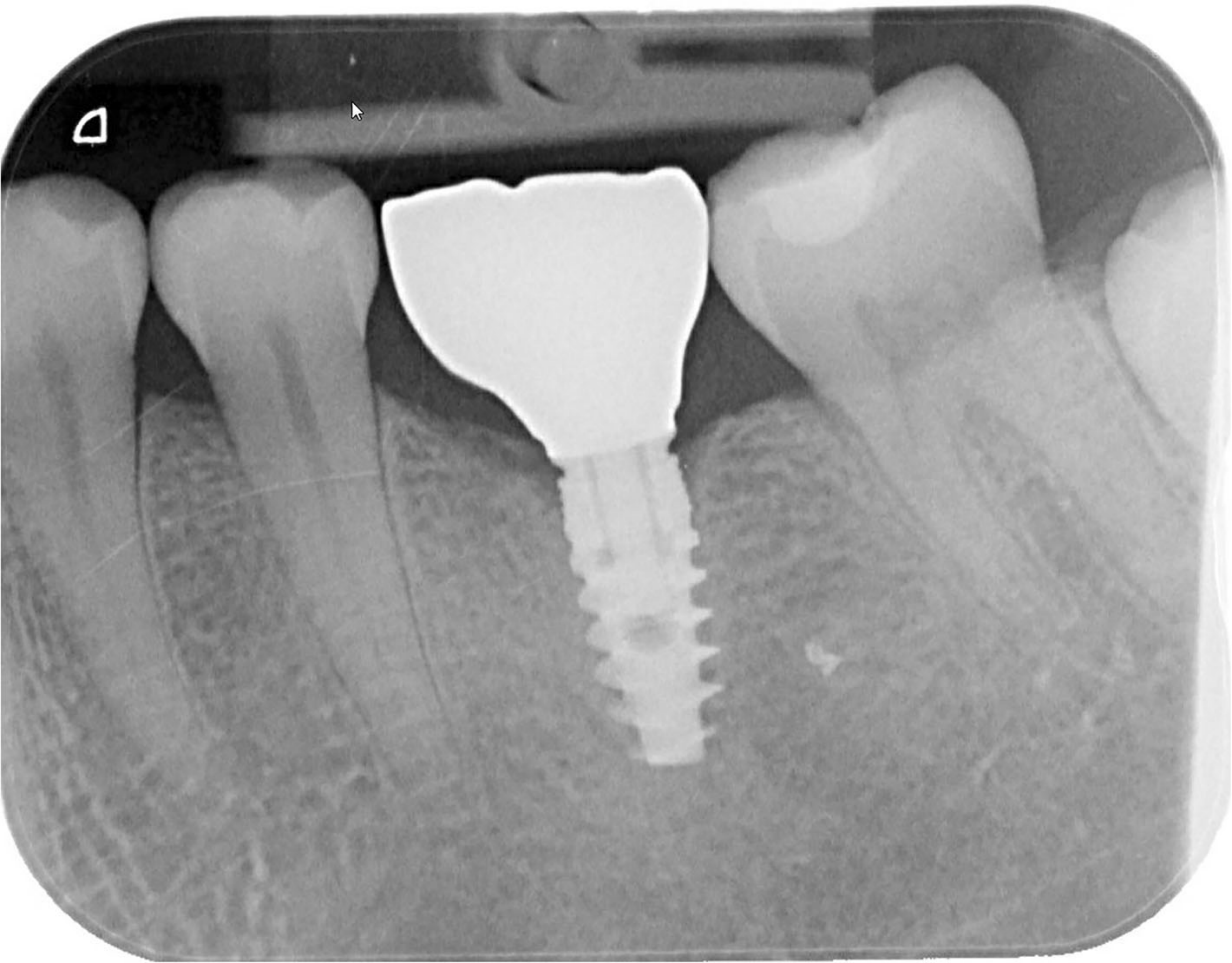
Intraoral radiograph of an immediately placed post-extractive dental implant with a full contour zirconia restoration after 1 year (same patient as in Fig. 1)

An impression was made at implant level 2 weeks after the second stage of the surgery in order to fabricate a single crown. A definitive full-zirconia crown (yttria-stabilized zirconium oxide) with an angulated screw channel (NobelProcera FCZ Implant Crown, NobelBiocare AB) was manufactured in the determined colour at a centralized milling facility (NobelProcera Service Center, Mahwah, NJ, USA) and then stained and glazed at a dental laboratory to attain the final colour (Ceram Essence and Ceram Glaze Paste, Ivoclar Vivadent, Schaan, Liechtenstein). The adapter and crown were assembled and screw-retained onto the implant with a torque of 35Ncm. The screw access hole was sealed with a cotton pellet and light-curing composite material (Fig. 2). The design of the occlusal surface allowed functional loading of restoration and implant. Immediately after placing the restoration, thorough oral hygiene instructions were given to all the patients.

### Outcome measures

Clinical and radiographic evaluations were performed 1 month and 1 year after restoration placement. The following criteria were considered:

- Implant survival. The survival rate of the implant was assessed 1 year after definitive restoration placement. An implant was defined as a failure when it was deemed necessary to remove the implant because of implant mobility as a consequence of loss of osseointegration;
- The marginal bone level as measured on standardized intraoral radiographs;
- Assessment of plaque accumulation with the modified Plaque Index [16];
- Assessment of bleeding tendency with the modified Sulcus Index [16];
- Assessment of peri-implant inflammation with the Gingival Index [17];
- Presence of calculus
- Probing pocket depth: measured to the nearest millimetre using a manual periodontal probe (Williams-Sulcus color-coded probe, Hu-Friedy, Chicago, IL, USA). The peri-implant sulcus was probed at four sites (at the mesial, distal, buccal and lingual/palatal side);
- Restoration survival;
- Complications related to the restoration;
- Patients’ satisfaction. Patients were asked to complete a questionnaire 1 year after restoration placement;
- Success rate: calculated from the criteria of success as proposed by Albrektsson and colleagues in 1986 [18].

Radiographs were taken and evaluated 1 month and 12 months after restoration placement using a parallel technique, with an X-ray holder for periapical radiographs. They were analysed using a specially designed computer software to perform linear measurements on digital radiographs. The calibration was carried out in the vertical plane of each radiograph by using the known distance of the implant and the distance of several threads in order to ensure correct measurements [19]. Crestal bone changes were determined by measuring, both mesially and distally, the distance from the implant reference point (the neck of the implant) to the margin level of the crestal bone. Bone loss was presented as the worst distal and/or mesial bone level change between 1 month and 12 months after restoration placement.

The patients’ recorded their satisfaction by means of filling out a questionnaire 1 year after restoration placement. The questionnaire included questions or statements to be answered on a 5-point rating scale ranging from “very dissatisfied” and “not in agreement” (score 1) to “very satisfied” and “in agreement” (score 5). The addressed topics were related to aesthetics and appearance, function (eating), sense (“feels like a natural tooth”), speech, and self-esteem. Furthermore, the patients were asked to mark their overall satisfaction with their dental situation at the 1-year evaluation on a 10-point rating scale from 0 to 10, whereby 10 is the highest satisfaction score.

### Statistical analysis

One observer was responsible for the collection and analysis of all the data. The worst score of the clinical and radiographic parameters evaluated per implant was used in the data analysis. Data were presented as frequencies without statistical testing.

## Results

All 15 consecutive patients eligible to join the study on the basis of the inclusion and exclusion criteria agreed to participate in this study. The patient characteristics are depicted in Table 1. All implant-supported restorations had natural antagonistic teeth. Four of the 15 patients treated had a mobile implant, which had to be removed (in two patients 3 months after crown placement and in two patients 6 months after crown placement). Of the four failing implants, two were placed in the mandible and two in the maxilla, three were positioned in between two natural teeth and one free-ending, and two of the original teeth in the site of implant loss were lost because of crown fracture and two because of root fracture. The remaining 11 patients completed the 1-year evaluation. Implant and restoration survival were 73.3% at the 1-year evaluation. All the failed implant patients were successfully treated again after a healing period of 3 months (delayed placement).

**Table 1:** Baseline characteristics of the study group

Mean age in years (sd, minimum-maximum)	57 (6.6, 44–67)
Gender (number male/female)	6/9
Reason of failure (severe caries/crown fracture/root fracture)	3/6/6
Implant position (maxilla/mandible)	7/8
Implant position (in between teeth/no tooth distally)	11/4

The mean scores of the indices for plaque, calculus, gingiva, and bleeding were very low, hence favourable (Table 2). There was no plaque and calculus at any of the restoration surfaces and no infection present as expressed with the gingival index. Some minor bleeding on probing was present in only one patient. The mean probing depth was 1.9 mm (SD 0.8 mm) at the 1-year follow-up. The mean marginal bone level at the 1-month evaluation session (T_1_) was 0.94 ± 0.54 mm apically of the neck of the implant (Table 3). The mean loss of marginal bone between 1 month after restoration placement (T_1_) and 1-year post-loading (T_12_) was 0.17 ± 0.73 mm (Table 4). One patient’s restoration became loose, which could be solved by retightening the screw. No other complications occurred during the 1-year evaluation period. The questionnaire revealed that only one patient evaded eating with the implant-supported restoration and that all patients were satisfied with colour and form of crown and surrounding mucosa. The patients mean overall satisfaction was 9.0 ± 0.6 from a scale of 1 to 10 at the 1-year evaluation (Table 5). Success rate, as calculated from the criteria of success as proposed by Albrektsson et al. [18], was 73.3%.

**Table 2:** Frequencies and percentages of plaque index scores (possible score 0–3), calculus index scores (possible score 0–1), gingival index scores (possible score 0–3), bleeding index scores (possible score 0–3), and mean value and standard deviation of probing depth (in mm) 1 month after restoration placement (T_1_) and after 1 year (T_12_)

	T_1_ (*n* = 15)	T_12_ (*n* = 11)
Plaque index	Score 0, 15 (100%)	Score 0, 11 (100%)
Calculus index	Score 0, 15 (100%)	Score 0, 11 (100%)
Gingival index	Score 0, 15 (100%)	Score 0, 11 (100%)
Bleeding index	Score 0, 15 (100%)	Score 0, 10 (91%) Score 1, 1 (9%)
Probing depth in mm (sd)	2.0 (0.9)	1.9 (0.8)

**Table 3:** Mean value, standard deviation, and frequency distribution (percentages) of marginal bone level at 1 month after restoration placement (T_1_)

Bone level (mm)	*n* = 15
Mean (SD)	− 0.94 mm (0.54)
> − 2.5 to − 2.0	1 (6.7)
> − 2.0 to − 1.5	2 (13.3)
> − 1.5 to − 1.0	4 (26.7)
> − 1.0 to − 0.5	5 (33.3)
> − 0.5 to 0.0	3 (20.0)

**Table 4:** Mean value, standard deviation, and frequency distribution (percentages) of marginal bone change between 1 month after restoration placement (T_1_) and 1 year in function (T_12_)

Bone change (mm)	*n* = 11
mean (SD)	− 0.17 mm (0.73)
> − 2.0 to − 1.5	1 (9.1)
> − 1.5 to − 1.0	0 (0.0)
> − 1.0 to − 0.5	2 (18.2)
> − 0.5 to 0.0	4 (36.4)
> 0.0 to 0.5	2 (18.2)
> 0.5 to 1.0	2 (18.2)

**Table 5:** Patient’s satisfaction 12 months (T_12_) after restoration placement

	Agreement percentage (*n* = 11)
Presence of shame	0.0
Self-confidence has decreased	0.0
Evades eating with the implant	9.1
The ability to chew has decreased	0.0
Implant influences speech	0.0
Implant influences taste	0.0
Not satisfied with the colour of the crown	0.0
Not satisfied with the form of the crown	0.0
Not satisfied with the colour of the mucosa around the crown	0.0
Not satisfied with the form of the mucosa around the crown	0.0
Overall satisfaction (possible score 0–10)	9.0 ± 0.6

## Discussion

Immediate placement of regular diameter implants in molar post-extraction sites of the maxilla and mandible resulted in a high implant failure rate during a 1-year follow-up period.

The implant survival rate was 73.3% after 1 year in function. The performance of immediate placements in post-extraction sites was also analysed in the Cafiero et al., Atieh et al., Tallarico et al., and Checchi et al. prospective 1-year studies [15, 20–22]. They reported a 1-year implant survival rate of 100%, 66.7%, 100%, and 89.4%, respectively. In the present study, an implant diameter of 4.3 mm was used in all the patients; the aforementioned studies used implant diameters of 4.8 mm, 8–9 mm, 7 mm, and 6–8 mm, respectively. The Atieh et al. [15] implant survival rate was the lowest (66.7%) and comparable with the survival rate in the present study, but it must be mentioned that the implant placement was combined with immediate provisionalization, whereas in the other studies, the implants were restored after 3–6 months. It was reported that implant stability in healed bone in the early postoperative period is positively influenced by the macro-thread design. A large thread depth with sharp thread edges and a small thread pitch (distance between two threads) gives higher implant stability than a small thread depth with v-shaped edges and large thread pitch [23, 24]. Finding enough primary stability in molar post-extraction sites may be difficult because of the thin interradicular bony septum. However, it is claimed that wide and ultra-wide diameter implants can be used in post-extraction molar sites to overcome this lack of primary stability [14]. All the failed implants of the present study became mobile within 6 months after functional loading. It can be assumed that, notwithstanding the initial primary stability of more than 45 Ncm, impaired healing with too less initial contact between implant and bone was the main reason for the failures. In the present study, implants were used implants with a large thread depth, sharp thread edges, but with a regular thread pitch and a regular diameter. The Cafiero et al., Tallarico et al., and Checchi et al. prospective 1-year delayed loading studies [20–22] used wide or ultra-wide diameter implants with a small thread pitch. This difference might be the reason for the much lower implant survival rate in the present study.

The mean marginal bone level was 0.94 mm below the neck of the implant at 1 month after restoration placement (T_1_). The optimal position of the peri-implant bone after a maturation period should be at the same level as the neck of the implant. This means that part of the biological width of the present study, which is acting as a barrier, was in contact with the implant surface roughness and was therefore more prone to biofilm formation, soft tissue infection, and peri-implantitis. Apparently, the large gap between the socket wall and the regular diameter implant, notwithstanding the local augmentation procedure, did not fill completely during healing which led to a compromised bone level. The Checchi et al. [22] study also mentioned that the bone level at the commencement of loading was 0.43 mm apically of the implant neck, possibly confirming the idea that better initial bone levels are reached with wider implants.

The mean change of the marginal bone height during the 1-year follow-up was − 0.17 mm, which is very limited. The Tallarico et al. and Checchi et al. studies’ peri-implant bone loss [21, 22] was 0.23 mm and 0.68 mm respectively, from initial loading to the 1-year evaluation. Apparently, after the period of healing and maturation peri-implant, bone levels remain rather stable.

Zirconia restorations are presumed to be highly biocompatible and can potentially attach to soft-tissue. It is claimed that zirconia promotes the attachment of human gingival fibroblasts in vivo, which is desirable because it mimics tooth cementum’s ability to attach to gingiva, forming the junctional epithelium [25]. With respect to the evaluation items of the peri-implant soft tissues of the present study, the findings are consistent with a healthy status, confirming the high biocompatibility of the material. The limited probing depth (mean value of 1.9 mm at the 1-year evaluation) is possibly associated with the claimed soft-tissue attachment potential. An advantage of screw-retained restorations is the absence of a microgap at the interface of the crown and abutments and the absence of possible cement remnants in the area of the peri-implant soft tissues. The use of abutments with angulated screw channels could, as a consequence of its design, promote soft tissue health. In addition, the high patient compliance to the prescribed post-treatment oral hygiene instructions could have played an important role in the observed very healthy peri-implant soft tissues.

In an attempt to incorporate the concept of patient engagement, this study investigated the patients’ satisfaction with the rehabilitated posterior region by assessing specific patient-centred outcomes. This was done by the patients filling out the established questionnaire 1 year after restoration placement [26, 27]. All the questioned outcome measures showed high patient satisfaction which is similar to the level reported in comparable studies with single tooth replacements in the posterior region and using the same questionnaire [26, 27]. Success rate in the present study, being 73.3%, was comparable with the calculated success rate in the study of Atieh et al. [15], being 66.7%.

A limitation of the pilot study is the small sample size. Nevertheless, the results are worthwhile mentioning. As to whether wider implants, with a small thread pitch, would have given better results within this study protocol is still under debate and should be explored with a larger study population. In addition, the inherent lack of a control group associated with a case series study is another factor that needs to be taken into account. Furthermore, even though the 1-year follow-up period is enough to indicate early implant failures and short-term restorative complications, it is considered to be a short post-treatment evaluation period.

## Conclusion

Within the limitations of this study, it has been demonstrated that immediate placement of regular diameter implants in molar post-extraction sites of maxilla and mandible resulted in a high implant failure rate during a 1-year follow-up period.

## Data Availability

The datasets used and/or analysed during the current study are available from the corresponding author on reasonable request.
